# Dietary Supplementation With Chlorogenic Acid Derived From *Lonicera macranthoides Hand-Mazz* Improves Meat Quality and Muscle Fiber Characteristics of Finishing Pigs via Enhancement of Antioxidant Capacity

**DOI:** 10.3389/fphys.2021.650084

**Published:** 2021-04-20

**Authors:** Wenlong Wang, Chaoyue Wen, Qiuping Guo, Jianzhong Li, Shanping He, Yulong Yin

**Affiliations:** ^1^Hunan Provincial Key Laboratory of Animal Intestinal Function and Regulation, Hunan International Joint Laboratory of Animal Intestinal Ecology and Health, Laboratory of Animal Nutrition and Human Health, School of Life Sciences, Hunan Normal University, Changsha, China; ^2^National Engineering Laboratory for Pollution Control and Waste Utilization in Livestock and Poultry Production, Hunan Provincial Key Laboratory of Animal Nutritional Physiology and Metabolic Process, Key Laboratory of Agro-ecological Processes in Subtropical Region, Institute of Subtropical Agriculture, Chinese Academy of Sciences, Changsha, China; ^3^University of Chinese Academy of Sciences, Beijing, China

**Keywords:** meat quality, myogenesis, chlorogenic acid, antioxidant capacity, finishing pig, muscle fiber characteristics

## Abstract

Chlorogenic acid (CGA), one of the most abundant polyphenol compounds in nature, is regarded as a potential feed additive to promote animal health and enhance the meat products’ quality via its various biological properties. The current study aims: (1) to determine whether dietary CGA supplementation improves meat quality and muscle fiber characteristics, and (2) to ascertain whether the corresponding improvement is associated with enhancing the antioxidant capacity of the finishing pigs. Thirty-two (Large × White × Landrace) finishing pigs with an average initial body weight of 71.89 ± 0.92 kg were allotted to 4 groups, and each was fed diets supplemented with 0, 0.02, 0.04, or 0.08% (weight/weight) of CGA. The meat quality traits, muscle fiber characteristics, and the serum and muscle antioxidant capacity were assessed. Results suggested that, compared with the control group, dietary CGA supplementation at a level of 0.04% significantly decreased the b^∗^ value and distinctly increased the inosinic acid content of longissimus dorsi (LD) and biceps femoris (BF) muscles (*P* < 0.01). Moreover, dietary supplementation with 0.04% of CGA markedly improved the amino acid composition of LD and BF muscles, as well as augmented the mRNA abundance of Nrf-2, GPX-1, MyoD, MyoG, and oxidative muscle fiber (I and IIa) in LD muscle (*P* < 0.05). This result indicates that a diet supplemented with 0.04% of CGA promotes myogenesis and induces a transformation toward more oxidative muscle fibers in LD muscle, subsequently improving meat quality. Besides, dietary supplementation with 0.02% and 0.04% of CGA notably enhanced the serum GSH-PX level (*P* < 0.01). Considering all these effects are closely related to the alteration of antioxidant activities of the finishing pigs, the underlying metabolism is likely connected to the boosting of their antioxidant capacity induced by dietary CGA.

## Introduction

With the improvement of living standards, there is an increasing demand for high-quality food products (Adebo, 2020). Many factors influence animal health and output in practical production, such as feed, drinking water, illness, environment, and management. Among them, feed including additives is an essential approach for a worldwide range of technologies to address the issue. Natural plant products are widely used as feed additives to ameliorate stress during the production process (Liu et al., 2019), ensuring good animal welfare, production efficiency, and animal products quality. It has become the focus of intense study to use different kinds of natural substances and herbal plant extracts as candidates for additives in recent years.

Chlorogenic acid (CGA) has been reported to exhibit various biological properties, including antioxidant (Liang and Kitts, 2015; Zhou et al., 2016b; Agunloye et al., 2019), anti-inflammatory (Vukelic et al., 2018), antibacterial (Wang et al., 2015), antiviral (Karar et al., 2016), hypoglycemic (Bassoli et al., 2008), and hypolipidemic (Sung et al., 2015) activities, which results in a broad scope in applications in the fields of animal production and human health care (Jiang and Xiong, 2016; Naveed et al., 2018; Ho et al., 2020). Hence, CGA is considered a potential feed additive to promote animal health benefits and meat products’ quality (Jiang and Xiong, 2016). Furthermore, as one of the most abundant polyphenol compounds, CGA is extensively distributed in nature. In China, especially in Hunan province, *Flos lonicerae* (*F. lonicerae*) and *Eucommia ulmoides* (*E. ulmoides*) are the two crucial CGA resources. The Longhui and Zhangjiajie districts in Hunan province are the main producing regions of *Lonicera macranthoides* (one species of *F. lonicerae; L. macranthoides*) and *E. ulmoides*, respectively. So far, they have been commonly used in the fields of medicine, tea, and food. Unfortunately, a mass of low-quality products and CGA-rich extract residuals are abandoned and consequently contribute to serious social problems, such as the waste of natural resources and environmental pollution. Using these underutilized parts of the natural resources as animal feed supplements can be a constructive solution.

Previous studies have indicated that *E. ulmoides* and its CGA-rich extracts supplemented in feeds have beneficial effects on growth performance, health status, and quality of animal products (Liu et al., 2018; Zhao et al., 2019). Comparatively, there is much less information on the effects of *L. macranthoides* and its extracts on animal production. Most relevant reports are concentrated on the effects of other CGA-rich plant extracts on animal growth performance or intestinal development and barrier functions of young animals (Chen et al., 2018; Zhang et al., 2018). To date, few studies have investigated the effects of dietary CGA supplementation on the quality of the animal product in any systematic way, let alone using a finishing pig model. The exact mechanisms by which dietary CGA supplementation may alter growth performance and animal products’ quality remain not fully understood. Therefore, it is of great significance to reveal the action mechanism of CGA, thus tapping the potential of local resources and promoting local economic development. Based on this, we hypothesized that dietary CGA supplementation might be attributed to the improvement of meat quality and muscle fiber characteristics of finishing pigs by influencing their antioxidant capacity. This study aimed to test this hypothesis, thereby elucidating the mechanisms by which CGA operates and its practical applications.

## Materials and Methods

### Animals and Treatments

The Animal Care Committee of the Institute of Subtropical Agriculture, Chinese Academy of Sciences (Changsha, China) approved the experimental design and all animal procedures. Thirty-two (Large × White × Landrace) barrows, with initial body weight (BW) of 71.89 ± 0.92 kg, were selected and fed a corn and soybean meal-based diet according to the recommendation of the National Research Council (NRC, 2012) for 5 days. Then they were randomly assigned to four groups with eight pigs (replicates) in each and fed with a basal diet supplemented with 0, 0.02, 0.04, or 0.08% of CGA (control group, 0.02% group, 0.04% group, 0.08% group; shown in Table 1). CGA used in the present study, provided by Changsha E.K Herb Co., Ltd., is a purified extract product (>98%) derived from *L. macranthoides* in Longhui county of Hunan province. During the 35-day experimental period (including a 5-day adjustment period), all the pigs were given *ad libitum* access to the diets and clean drinking water. Feed intake was recorded daily. At the start and the end of this experiment, all pigs were weighed after overnight fasting.

**TABLE 1:** Composition and nutrient levels of the experimental diets (air-dry basis, %).

Items	Dietary levels of CGA^1^			
	
	0 (Control)	0.02%	0.04%	0.08%
**Ingredient composition,%**				
Corn	67.00	66.98	66.95	66.90
Soybean meal	23.76	23.76	23.77	23.78
Wheat bran	6.00	6.00	6.00	6.00
Soybean oil	0.88	0.88	0.88	0.08
CGA	0	0.02	0.04	0.08
Lysine	0.01	0.01	0.01	0.01
Dicalcium phosphate	0.50	0.50	0.50	0.50
Limestone	0.55	0.55	0.55	0.55
Salt	0.30	0.30	0.30	0.30
1% Premix^2^	1.00	1.00	1.00	1.00
Total	100.00	100.00	100.00	100.00
**Nutrient levels^3^, %**				
Digestible energy (MJ/kg)	14.21	14.21	14.20	14.20
Crude Protein^4^	16.02	16.00	16.01	16.02
Lysine	0.73	0.73	0.73	0.73
Methionine + Cysteine	0.51	0.51	0.51	0.51
Threonine	0.52	0.52	0.52	0.52
Tryptophan	0.17	0.17	0.17	0.17
Total calcium^4^	0.50	0.50	0.51	0.49
Total Phosphorus^4^	0.46	0.45	0.44	0.45
Available phosphorus	0.19	0.19	0.19	0.19

*^1^CGA, chlorogenic acid; the same as below.*

*^2^Supplied per kg of diet: CuSO_4_⋅5H_2_O 19.8 mg; KI 0.20 mg; FeSO_4_⋅7H_2_O 400 mg; NaSeO_3_ 0.56 mg; ZnSO_4_⋅7H_2_O 359 mg; MnSO_4_⋅H_2_O 10.2 mg; Vitamin K (menadione) 5 mg; Vitamin B_1_ 2 mg; Vitamin B_2_ 15 mg; Vitamin B_12_ 30 μg; Vitamin A 5,400 IU; Vitamin D_3_ 110 IU; Vitamin E 18 IU; Choline chloride 800 mg; Antioxidants 20 mg; Fungicide 100 mg.*

*^3^Calculated values.*

*^4^Measured values.*

### Sample Collection

At the end of the feeding trial, blood samples from overnight fasting (12 h) pigs were collected into 10 mL tubes by inferior vena cava puncture to determine serum biochemical parameters. All blood samples were allowed to clot at room temperature. The serum was then separated immediately by centrifugation (3,000 × *g*, 10 min, 4°C) and stored at −80°C until use. Pigs were slaughtered via electrical stunning (250 V, 0.5 A, for 5–6 s) and then exsanguinated and eviscerated. Subsequently, carcasses were weighed and split longitudinally. The longissimus dorsi (LD) and biceps femoris (BF) muscle samples were rapidly excised from the right side of the carcass. Samples from LD and BF muscles were immediately frozen in liquid nitrogen and then stored at −80°C until further analysis.

### Meat Quality Analysis

The meat quality was determined using LD muscle samples, with no external fat or connective tissue. The meat color was measured at 45 min postmortem with a portable chromameter (CR410, Konica Minolta Sensing, Inc., Tokyo, Japan), which was calibrated with a white tile according to the manufacturer’s manual. Mean L^∗^(lightness), a^∗^(redness), and b^∗^(yellowness) values were collected from three different locations on the cut surface of each sample. Measurements of pH were collected at 45 min (pH_45 min_) and 24 h (pH_24 h_) postmortem using a pH-meter (pH-Star, Matthäus, Pöttmes, Germany), previously calibrated with pH 4.6 and 7.0 buffers. The drip loss was determined at 24 h postmortem. Briefly, a muscle section was cut lengthwise with the direction of the fiber (2 × 3 × 5 cm), trimmed of fat, weighed, suspended in a polyethylene plastic cup (ensuring that the sample did not contact the bag), and then stored at 4°C. After 24 h, the sample was reweighed to calculate drip loss percentage. Meat samples of approximately 150 g were weighed and placed in plastic bags and then cooked in an 80°C water bath until the central temperature reached 70°C. Following cooling to room temperature, the samples were weighed to calculate cook loss. Afterward, the cooked samples were immediately cut (parallel to the direction of the muscle fiber) into shaped strips (1.27 cm diameter, 3.0 cm length) for meat tenderness measurement using an advanced texture analyzer (TMS/PRO, Food Technology Corporation, Sterling, VA, United States). At least six replicates of each sample were measured to calculate the average value. Additionally, marbling was scored by recording a mean value of scores assessed by three people based on NPCC Official marbling quality standards (Berg, 2000).

### Muscle Nutritional Components Analysis

Each muscle sample (20–30 g) was sliced up and weighed. It was reweighed after placement in a clean weighing bottle. Subsequently, the weighing bottle was placed into a freeze dryer (Alpha 2-4 LD plus, CHRIST, Osterode, Germany) with a temperature of −50°C for 72 h and then reweighed. The difference in weight between the initial and dried samples was used to calculate the dry matter percentage. After that, the dried samples were pulverized (freeze-dried muscle sample powder) to analyze crude protein (CP). Measurement of the CP content was performed following the methods of the Association of Analytical Communities (AOAC, 2006).

Contents of inosinic acid were determined using a High-Performance Liquid Chromatography method. Briefly, 0.2 g freeze-dried muscle sample powder was added to 5 mL perchloric acid (HClO_4_, 0.6 mol/L), and the mixture was homogenized with an ice bath in an ultrasonic cleaning machine. Afterward, the mixture was centrifuged (6,000 × *g*, 10 min, 4°C) for deproteinization. The volume of supernatant was then made up to 10 mL with double-distilled water. Half of the mixture was adjusted to pH 6.9–7.1 using a potassium hydroxide (KOH, 1 mol/L) and perchloric acid (HClO_4_, 0.6 mol/L) solution, allowed to stand for 10 min in an ice bath, and filtered through a 0.22 mm microfilter to remove potassium perchlorate. The filtrate was adjusted to a total volume of 10 mL with distilled water, and it was analyzed by high-performance liquid chromatography (1260 Infinity II, Agilent, Santa Clara, CA, United States). The chromatographic conditions were as follows: the reversed-phase chromatographic column (Agilent ZORBAX Eclipse XDB-C18; 4.6 mm × 250 mm, 5 μm), with a mobile phase of phosphate buffer solution (100%) at a flow rate of 1.0 mL/min, was used in this chromatographic assay. The injection volume, column temperature, and UV detection wavelength were 10 μL, 25°C, and 210 nm, respectively.

### Measurement of Serum Antioxidant Parameters

Serum antioxidant parameters were determined using commercially available assay kits for superoxide dismutase (SOD, #A001; WST-1 method), reduced glutathione (GSH, #A006; microplate method), glutathione peroxidase (GSH-PX, #A005; colorimetric method), malondialdehyde (MDA, #A003; TBA method) and total antioxidant capacity (T-AOC, #A015; ABTS method), respectively (Nanjing Jiancheng Biotech, Nanjing, China).

The determination of the SOD activity relied on the principle that the superoxide anion reduces water-soluble tetrazolium (WST), producing yellow formazan, which can be measured using a spectrophotometer at 450 nm. Antioxidants within the samples inhibit yellow WST formation. The formazan formation rate was converted to SOD activity for a specific concentration of protein.

GSH reacts with dithiodinitrobenzoic acid (DTNB) to produce a yellow compound, which can be colorimetrically determined at 405 nm to determine the content of GSH quantitatively. GSH-PX was measured by the enzymatic reaction, which was initiated by adding H_2_O_2_ to the reaction mixture containing GSH. GSH-PX can promote the reaction of H_2_O_2_ with GSH to generate H_2_O and oxidized glutathione (GSSG). The activity of GSH-PX can be expressed by the speed of this reaction and thus obtained by measuring the consumption of GSH in this enzymatic reaction.

MDA levels were analyzed by a method based on the reaction with thiobarbituric acid (TBA) at >95°C. In the TBA test reaction, MDA or MDA-like substances and TBA condense together to form a pink pigment with a maximum absorption peak at 532 nm.

The essentials of T-AOC determination were based on ABTS [2, 2′-azino-bis(3-ethylbenzothiazoline-6-sulfonic acid)] method. The ABTS can be oxidized to green ABTS^⋅+^ under the action of oxidants. In the presence of antioxidants, the production of ABTS^⋅+^ will be inhibited. The absorbance of ABTS^⋅+^ was measured at 405 nm or 734 nm to determine and calculate the T-AOC.

### Hydrolyzed Amino Acid Content Analysis

Hydrolytic amino acids of the LD and BF muscles were identified by an amino acid analyzer (L8800, Hitachi, Tokyo, Japan). Approximately 0.2 g of freeze-dried muscle sample powder was hydrolyzed with 10 mL of hydrochloric acid solution (HCl, 6 mol/L) in a sealed ampoule bottle for 22 h at 110 (±1)°C. All the hydrolyzates were diluted with double-distilled water in a volumetric flask of 50 mL, and 1 mL of the hydrolyzate diluent was passed through a 0.22 mm membrane filter. Samples were analyzed for amino acid content by comparing peak profiles of the obtained samples with standard amino acid profiles.

### Real-Time PCR

Total RNA was isolated from muscle samples using the TRIzol reagent (Invitrogen, Carlsbad, CA, United States). The quality and quantity of RNA were determined via ultraviolet spectroscopy using a NanoDrop ND-1000 (Thermo Fisher Scientific, Wilmington, DE, United States). The A260/280 ratios were between 1.9 and 2.1, and RNA integrity was evaluated using electrophoresis with 1.5% agarose and ethidium bromide staining (1.25 ng/μL; Sigma-Aldrich, St. Louis, MO, United States). After that, approximately 1 μg of total RNA was incubated with DNase I (Fermentas, WI, United States) followed by reverse transcription using a First-Strand cDNA Synthesis Kit (Promega, Madison, WI, United States) according to the manufacturer’s protocol. The cDNA synthesized with Oligo dT and superscript II reverse-transcriptase was stored at −80°C before further processing. The primer sequences for selected genes are listed in Table 2. The relative expressions of the target genes were determined by real-time PCR performed using a LightCycler 480 II PCR system (Roche Life Science, Basel, Switzerland). Real-time PCR was performed duplicated for each cDNA sample, using SYBR Green I as a PCR core reagent in a final volume of 20 μL. PCR conditions were as follows: incubation for 10 min at 95°C, followed by 40 cycles of denaturation for 15 s at 95°C, then annealing and extension for 60 s at 56–64°C. The housekeeping gene β-actin was used as an internal control to normalize the expression of target genes. mRNA expression levels of the target genes, expressed as arbitrary units, were acquired from the value of the threshold cycle (Ct) of real-time PCR relative to β-actin using the comparative 2^–ΔΔ*Ct*^ method.

**TABLE 2:** Primers used for Real-time PCR analysis.

Genes^#^	Primers	Sequences (5′-3′)	Size, bp
SOD1	Forward	GAGACCTGGGCAATGTGACT	189
	Reverse	CCAAACGACTTCCAGCATTT	
Nrf-2	Forward	GAAAGCCCAGTCTTCATTGC	190
	Reverse	TTGGAACCGTGCTAGTCTCA	
GPX1	Forward	AGCCCAACTTCATGCTCTTC	159
	Reverse	CATTGCGACACACTGGAGAC	
MyHC I	Forward	AAGGGCTTGAACGAGGAGTAGA	114
	Reverse	TTATTCTGCTTCCTCCAAAGGG	
MyHC IIa	Forward	GCTGAGCGAGCTGAAATCC	136
	Reverse	ACTGAGACACCAGAGCTTCT	
MyHC IIb	Forward	CACTTTAAGTAGTTGTCTGCCTTGAG	83
	Reverse	GGCAGCAGGGCACTAGATGT	
MyHC IIx	Forward	AGCTTCAAGTTCTGCCCCACT	76
	Reverse	GGCTGCGGGTTATTGATGG	
MyoD	Forward	CAACAGCGGACGACTTCTATG	383
	Reverse	GCGCAAGATTTCCACCTT	
MyoG	Forward	GCAGGGTGCTCCTCTTCA	230
	Reverse	AGGCTACGAGCGGACTGA	
β-actin	Forward	TGCGGGACATCAAGGAGAAG	216
	Reverse	AGTTGAAGGTGGTCTCGTGG	

*^#^SOD1, superoxide dismutase 1; Nrf-2, nuclear factor E2-related factor-2; GPX1, glutathione peroxidase 1; MyHC, myosin heavy chain; MyoD, myogenic differentiation factor 1; MyoG, myogenin; β-actin, housekeeping gene.*

### Statistical Analysis

All data were analyzed by one-way ANOVA followed by Duncan’s multiple comparison test using the SAS 8.2 software (SAS Institute Inc., Cary, NC, United States). Results are presented as means ± SEM. Differences between significant means were considered statistically significant at *P* < 0.05 and displaying a tendency toward significance at *P* < 0.10. Graphs were made using the GraphPad Prism 7 software (GraphPad Software Inc., San Diego, CA, United States).

## Results

### Meat Quality

The meat quality indexes of finishing pigs fed with different levels of dietary CGA supplementation are exhibited in Table 3. No significant effect was detected with regard to L^∗^ value, a^∗^ value, pH_45 min_, pH_24 h_, drip loss, cooking loss, marble score, and meat tenderness. A diet supplemented with 0.04% of CGA resulted in a significantly decreased b^∗^ value (*P* < 0.01), but there was no statistical difference in the b^∗^ values between 0.02 and 0.04% CGA groups (*P* > 0.05). These results indicate that a diet supplemented with CGA at a level of 0.04% might improve meat quality by decreasing its yellowness.

**TABLE 3:** Effects of different levels of dietary CGA supplementation on meat quality of the finishing pigs.

Items^&^	Control	0.02%	0.04%	0.08%	*P*-value
L*	50.01 ± 0.38	49.35 ± 0.41	49.05 ± 0.73	50.17 ± 0.72	0.48
a*	12.82 ± 0.17	12.62 ± 0.44	13.52 ± 0.48	12.80 ± 0.28	0.33
b*	5.02 ± 0.09^*a*^	4.74 ± 0.07^*ab*^	4.38 ± 0.19^*b*^	5.12 ± 0.19^*a*^	<0.01
pH_45 min_	6.56 ± 0.05	6.49 ± 0.06	6.41 ± 0.05	6.46 ± 0.03	0.25
pH_24 h_	5.42 ± 0.04	5.40 ± 0.05	5.41 ± 0.07	5.37 ± 0.03	0.86
Drip loss, %	2.31 ± 0.06	2.58 ± 0.15	2.38 ± 0.31	2.81 ± 0.18	0.29
Cooking loss, %	28.28 ± 0.43	28.05 ± 0.29	28.91 ± 0.32	28.17 ± 0.24	0.27
Marbling score	2.17 ± 0.17	2.17 ± 0.31	2.33 ± 0.21	2.67 ± 0.21	0.39
Meat tenderness, N	68.31 ± 2.00	74.53 ± 3.64	66.43 ± 3.52	65.88 ± 2.45	0.16

^&^L*, lightness; a*, redness; b*, yellowness.

*^*a,b*^Values in the same row with different superscript letters are significantly different at *P* < 0.05 (The superscript letter a represents the maximum mean value and c represents the minimum. There is a significant difference between a and b, but no difference between a and ab, or b and ab. The same as below).*

### Serum Antioxidant Parameters

As shown in Table 4, compared with the control group, dietary supplementation with 0.02% of CGA significantly increased the serum GSH levels (*P* < 0.05). No difference was observed in the serum GSH levels between the 0.02 and 0.08% CGA groups (*P* > 0.05). Moreover, dietary supplementation with 0.02 and 0.04% of CGA significantly increased the serum GSH-PX level (*P* < 0.01). Compared with the 0.04 and 0.08% CGA groups, the serum GSH-PX level of the 0.02% CGA group increased 21.96 and 45.77%, respectively (*P* < 0.05). Meanwhile, there was no significant difference in the serum GSH-PX levels between the 0.04 and 0.08% CGA groups (*P* > 0.05). Additionally, dietary CGA supplementation had no effect on serum SOD, MDA, and T-AOC levels (*P* > 0.05).

**TABLE 4:** Effects of different levels of dietary CGA supplementation on serum antioxidant parameters of the finishing pigs.

Items^1^	Control	0.02%	0.04%	0.08%	*P*-value
SOD, U/mL	57.85 ± 4.69	63.38 ± 4.20	65.41 ± 2.17	58.30 ± 1.94	0.41
GSH, mg/L	4.60 ± 0.31^*b*^	6.40 ± 0.57^*a*^	3.85 ± 0.73^*b*^	5.30 ± 0.38^*ab*^	0.02
GSH-PX, U	517.76 ± 25.13^*c*^	853.64 ± 25.20^*a*^	699.95 ± 77.52^*b*^	585.60 ± 21.57^*bc*^	<0.01
MDA, nmol/mL	2.30 ± 0.28	1.85 ± 0.16	2.12 ± 0.20	2.74 ± 0.59	0.27
T-AOC, U/mL	0.79 ± 0.18	1.11 ± 0.18	0.92 ± 0.23	0.80 ± 0.12	0.60

*^1^SOD, superoxide dismutase; GSH, glutathione; GSH-PX, glutathione peroxidase; MDA, malonaldehyde; T-AOC, total-antioxidant capacity.*

*^*a,b,c*^Values in the same row with different superscript letters are significantly different at *P* < 0.05.*

### Muscle Nutritional Components

The contents of dry matter, crude protein, and inosinic acid were determined (Table 5) to evaluate the effects of dietary CGA supplementation on muscle nutritional components. Compared with the control group, dietary supplementation with CGA at a level of 0.04% significantly increased the inosinic acid content of LD and BF muscles (*P* < 0.01). However, dietary CGA supplementation did not overtly affect the crude protein content (*P* > 0.05). Compared with the control and the 0.08% CGA group, the 0.02% CGA group contained a significantly lower dry matter content within the BF muscle (*P* > 0.05). Thus, it was implied that dietary supplementation with CGA at a level of 0.04% might improve meat quality by increasing inosinic acid content.

**TABLE 5:** Effects of different levels of dietary CGA supplementation on muscle nutritional components of the finishing pigs.

Items	Control	0.02%	0.04%	0.08%	*P*-value
**Longissimus dorsi muscle**					
Dry matter, %	24.01 ± 0.16	24.54 ± 0.26	24.35 ± 0.46	24.59 ± 0.14	0.48
Crude protein, %	21.65 ± 0.19	21.85 ± 0.15	21.79 ± 0.19	21.91 ± 0.16	0.76
Inosinic acid, mg/g	1.40 ± 0.12^*b*^	2.26 ± 0.47^*a*^	2.59 ± 0.27^*a*^	1.23 ± 0.08^*b*^	<0.01
**Biceps femoris muscle**					
Dry matter, %	23.92 ± 0.09^*ab*^	23.35 ± 0.04^*c*^	23.52 ± 0.30^*bc*^	24.30 ± 0.05^*a*^	<0.01
Crude protein, %	21.24 ± 0.66	20.77 ± 0.22	20.13 ± 0.27	21.66 ± 0.58	0.16
Inosinic acid, mg/g	1.82 ± 0.13^*b*^	2.44 ± 0.21^*ab*^	3.31 ± 0.49^*a*^	1.59 ± 0.30^*b*^	<0.01

*^*a,b,c*^Values in the same row with different superscript letters are significantly different at *P* < 0.05.*

### The Content of Hydrolyzed Amino Acids in Skeletal Muscles

The contents of hydrolyzed amino acids in LD muscle are shown in Table 6. Dietary CGA supplementation did not affect the Leu, Met, Thr, Ser, and Tyr proportions (*P* > 0.05). Both 0.04 and 0.08% CGA groups contained greater proportions of EAA, FAA, NEAA, and TAA (*P* < 0.05), compared with the 0.02% CGA group. The 0.08% CGA group had the highest Arg proportion, which was significantly higher than that of the 0.02% CGA group (*P* < 0.05). Also, the 0.08% CGA group had the highest His and Phe proportions, significantly higher than those of all the other groups (*P* < 0.05). The 0.02% CGA group contained decreased Ile, Lys, Asp, and Glu proportions (*P* < 0.05), compared with the 0.04 and 0.08% CGA groups. The highest Val, Ala, and Gly proportions were observed in the 0.04% CGA group, significantly higher than those of the 0.02% CGA group (*P* < 0.05). The results above strongly indicate that dietary supplementation with CGA at a level of 0.02% risks reducing the contents of hydrolyzed amino acids in LD muscle. On the contrary, dietary supplementation with 0.04 or 0.08% of CGA may improve the nutritional value and meat quality of LD muscle.

**TABLE 6:** Effects of different levels of dietary CGA supplementation on the content of hydrolyzed amino acids in LD muscle of the finishing pigs (μg/100 mg).

Items	Control	0.02%	0.04%	0.08%	*P*-value
**Essential amino acids**					
Arg	5.57 ± 0.08^*ab*^	5.41 ± 0.06^*b*^	5.69 ± 0.08^*ab*^	5.86 ± 0.16^*a*^	0.03
His	4.74 ± 0.06^*b*^	4.72 ± 0.07^*b*^	4.79 ± 0.05^*b*^	5.18 ± 0.17^*a*^	<0.01
Ile	4.03 ± 0.07^*ab*^	3.82 ± 0.09^*b*^	4.13 ± 0.05^*a*^	4.19 ± 0.11^*a*^	0.02
Leu	6.95 ± 0.08	6.84 ± 0.06	7.09 ± 0.09	7.06 ± 0.14	0.25
Lys	8.48 ± 0.13^*a*^	7.96 ± 0.19^*b*^	8.53 ± 0.12^*a*^	8.71 ± 0.23^*a*^	0.02
Met	3.16 ± 0.06	3.19 ± 0.08	3.27 ± 0.09	2.97 ± 0.14	0.21
Phe	2.73 ± 0.06^*b*^	2.55 ± 0.09^*b*^	2.76 ± 0.05^*b*^	3.01 ± 0.09^*a*^	<0.01
Thr	4.42 ± 0.07	4.35 ± 0.04	4.57 ± 0.05	4.57 ± 0.11	0.10
Val	5.57 ± 0.14^*ab*^	5.41 ± 0.14^*b*^	5.97 ± 0.08^*a*^	5.75 ± 0.18^*ab*^	<0.05
EAA^1^	45.66 ± 0.69^*ab*^	44.25 ± 0.57^*b*^	46.49 ± 0.58^*a*^	47.31 ± 1.22^*a*^	0.05
**Non-essential amino acids**					
Ala	6.27 ± 0.15^*ab*^	6.06 ± 0.13^*b*^	6.70 ± 0.08^*a*^	6.35 ± 0.23^*ab*^	0.05
Asp	8.64 ± 0.15^*ab*^	8.15 ± 0.21^*b*^	8.80 ± 0.13^*a*^	9.11 ± 0.24^*a*^	<0.01
Cys	1.78 ± 0.10^*b*^	1.21 ± 0.09^*c*^	2.20 ± 0.15^*a*^	1.57 ± 0.10^*b*^	<0.01
Glu	12.75 ± 0.17^*a*^	12.20 ± 0.04^*b*^	12.95 ± 0.14^*a*^	12.90 ± 0.31^*a*^	0.03
Gly	4.10 ± 0.05^*ab*^	4.05 ± 0.02^*b*^	4.26 ± 0.06^*a*^	4.23 ± 0.10^*ab*^	0.08
Ser	3.73 ± 0.05	3.64 ± 0.05	3.79 ± 0.05	3.82 ± 0.10	0.23
Tyr	2.19 ± 0.02	2.27 ± 0.04	2.26 ± 0.05	2.27 ± 0.06	0.55
FAA^2^	37.33 ± 0.57^*ab*^	35.87 ± 0.37^*b*^	38.39 ± 0.45^*a*^	38.44 ± 1.01^*a*^	0.02
NEAA^3^	39.46 ± 0.59^*ab*^	37.57 ± 0.40^*b*^	40.94 ± 0.50^*a*^	40.25 ± 1.07^*a*^	<0.01
TAA^4^	85.12 ± 1.28^*ab*^	81.82 ± 0.96^*b*^	87.74 ± 1.07^*a*^	87.55 ± 2.29^*a*^	0.03

*^1^EAA, essential amino acids = Arg + His + Ile + Leu + Lys + Met + Phe + Thr + Val.*

*^2^FAA, flavor amino acids = Glu + Asp + Ala + Arg + Gly.*

*^3^NEAA, non-essential amino acids = Ala + Asp + Cys + Glu + Gly + Ser + Tyr.*

*^4^TAA, total amino acids.*

*^*a,b,c*^Values in the same row with different superscript letters are significantly different at *P* < 0.05.*

The contents of hydrolyzed amino acids in BF muscle are shown in Table 7. The 0.04% CGA supplementation resulted in the highest proportions of His, Ile, Leu, Lys, Phe, Thr, Val, EAA, Ala, Asp, Glu, Ser, FAA, NEAA, and TAA, significantly greater than those of the control and 0.08% CGA groups (*P* < 0.05). Additionally, the 0.02% CGA group had the lowest Met proportion and the hugest Cys proportion, but there was no difference with those of the 0.04% CGA group (*P* > 0.05). This part showed that dietary supplementation with both 0.02% and 0.04% of CGA enhanced the composition of amino acids in BF muscle. Overall, the most effective dose of dietary supplementation herein is 0.04%.

**TABLE 7:** Effects of different levels of dietary CGA supplementation on the content of hydrolyzed amino acids in BF muscle of finishing pigs (μg/100 mg).

Items	Control	0.02%	0.04%	0.08%	*P*-value
**Essential amino acids**					
Arg	4.70 ± 0.05^*c*^	5.08 ± 0.10^*b*^	5.49 ± 0.10^*a*^	4.58 ± 0.07^*c*^	<0.01
His	3.49 ± 0.10^*b*^	3.58 ± 0.07^*ab*^	3.89 ± 0.19^*a*^	3.50 ± 0.07^*b*^	0.07
Ile	3.42 ± 0.06^*b*^	3.61 ± 0.06^*ab*^	3.77 ± 0.13^*a*^	3.37 ± 0.05^*b*^	<0.01
Leu	6.18 ± 0.08^*bc*^	6.47 ± 0.09^*ab*^	6.55 ± 0.16^*a*^	6.13 ± 0.06^*c*^	0.01
Lys	6.98 ± 0.10^*b*^	7.47 ± 0.15^*a*^	7.62 ± 0.25^*a*^	6.71 ± 0.09^*b*^	<0.01
Met	2.12 ± 0.08^*a*^	1.88 ± 0.03^*b*^	2.04 ± 0.09^*ab*^	2.06 ± 0.06^*ab*^	0.09
Phe	2.31 ± 0.03^*b*^	2.61 ± 0.08^*a*^	2.65 ± 0.09^*a*^	2.26 ± 0.04^*b*^	<0.01
Thr	3.81 ± 0.05^*b*^	3.96 ± 0.06^*ab*^	4.16 ± 0.14^*a*^	3.75 ± 0.04^*b*^	<0.01
Val	3.93 ± 0.10^*b*^	4.10 ± 0.08^*ab*^	4.38 ± 0.18^*a*^	4.04 ± 0.05^*b*^	0.04
EAA^1^	36.93 ± 0.61^*bc*^	38.75 ± 0.57^*ab*^	40.55 ± 1.20^*a*^	36.41 ± 0.41^*c*^	<0.01
**Non-essential amino acids**					
Ala	4.64 ± 0.10^*b*^	5.02 ± 0.12^*a*^	5.11 ± 0.15^*a*^	4.68 ± 0.08^*b*^	<0.01
Asp	7.31 ± 0.12^*b*^	7.67 ± 0.13^*ab*^	8.06 ± 0.28^*a*^	7.23 ± 0.06^*b*^	<0.01
Cys	0.72 ± 0.04^*b*^	0.93 ± 0.08^*a*^	0.86 ± 0.07^*ab*^	0.89 ± 0.06^*ab*^	0.13
Glu	11.00 ± 0.16^*b*^	11.74 ± 0.23^*a*^	12.04 ± 0.34^*a*^	10.91 ± 0.09^*b*^	<0.01
Gly	3.60 ± 0.04^*b*^	3.89 ± 0.09^*a*^	3.76 ± 0.03^*ab*^	3.62 ± 0.09^*b*^	0.01
Ser	2.15 ± 0.05^*b*^	3.32 ± 0.05^*ab*^	3.48 ± 0.10^*a*^	3.13 ± 0.03^*b*^	<0.01
Tyr	2.29 ± 0.03^*a*^	2.23 ± 0.04^*a*^	2.26 ± 0.05^*a*^	2.10 ± 0.04^*b*^	<0.01
FAA^2^	31.25 ± 0.44^*b*^	33.41 ± 0.50^*a*^	34.47 ± 0.85^*a*^	31.03 ± 0.33^*b*^	<0.01
NEAA^3^	32.72 ± 0.47^*b*^	34.80 ± 0.53^*a*^	35.57 ± 0.95^*a*^	32.57 ± 0.32^*b*^	<0.01
TAA^4^	69.95 ± 1.07^*b*^	73.55 ± 1.07^*a*^	76.13 ± 2.14^*a*^	68.98 ± 0.70^*b*^	<0.01

*^1^EAA, essential amino acids = Arg + His + Ile + Leu + Lys + Met + Phe + Thr + Val.*

*^2^FAA, flavor amino acids = Glu + Asp + Ala + Arg + Gly.*

*^3^NEAA, non-essential amino acids = Ala + Asp + Cys + Glu + Gly + Ser + Tyr.*

*^4^TAA, total amino acids.*

*^*a,b,c*^Values in the same row with different superscript letters are significantly different at *P* < 0.05.*

### The Differential mRNA Expression of Antioxidant-Related Genes in Skeletal Muscles

**FIGURE 1:**
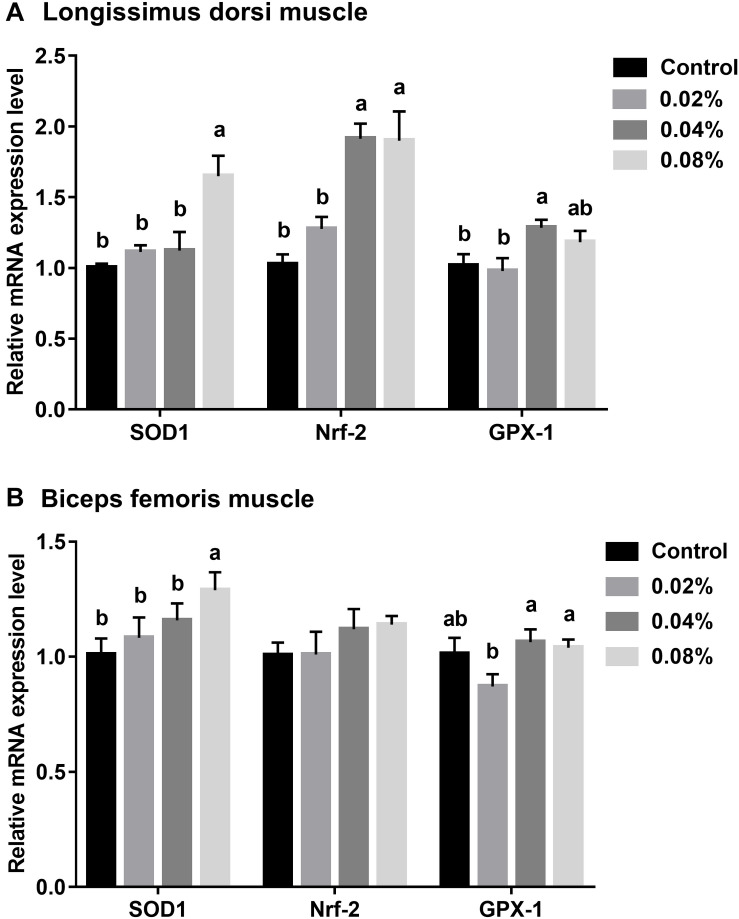
Effects of different levels of dietary CGA supplementation on relative mRNA expression levels of antioxidant-related genes in LD **(A)** and BF **(B)** muscles of finishing pigs. Values are means, with their standard errors represented by vertical bars. Values with different letters differ (*P* < 0.05).

The relative mRNA expression levels were normalized against β-actin gene expression levels. The relative expression levels of antioxidant-related genes in LD muscle are presented in Figure 1A. Dietary supplementation with 0.04% of CGA significantly increased Nrf-2 and GPX-1 mRNA expression levels (*P* < 0.05). When the supplementation dose increased to 0.08%, SOD1 and Nrf-2 mRNA expression levels were also significantly increased (*P* < 0.05). However, there was no difference in Nrf-2 and GPX-1 mRNA expression levels between the 0.04 and 0.08% CGA groups (*P* > 0.05).

The relative expression levels of antioxidant-related genes in BF muscle are stated in Figure 1B. Dietary supplementation with CGA at a level of 0.08% increased the SOD1 mRNA expression level (*P* < 0.05). Compared with the control group, dietary CGA supplementation did not significantly affect the GPX-1 mRNA expression (*P* > 0.05). The GPX-1 mRNA expression of the 0.02% CGA group was significantly lower than that of the 0.04% and 0.08% CGA groups (*P* < 0.05). Together, this suggests that dietary supplementation with 0.04% and 0.08% of CGA enlarges the antioxidant capacity of LD and BF muscles.

### Muscle Fiber Diameter

**FIGURE 2:**
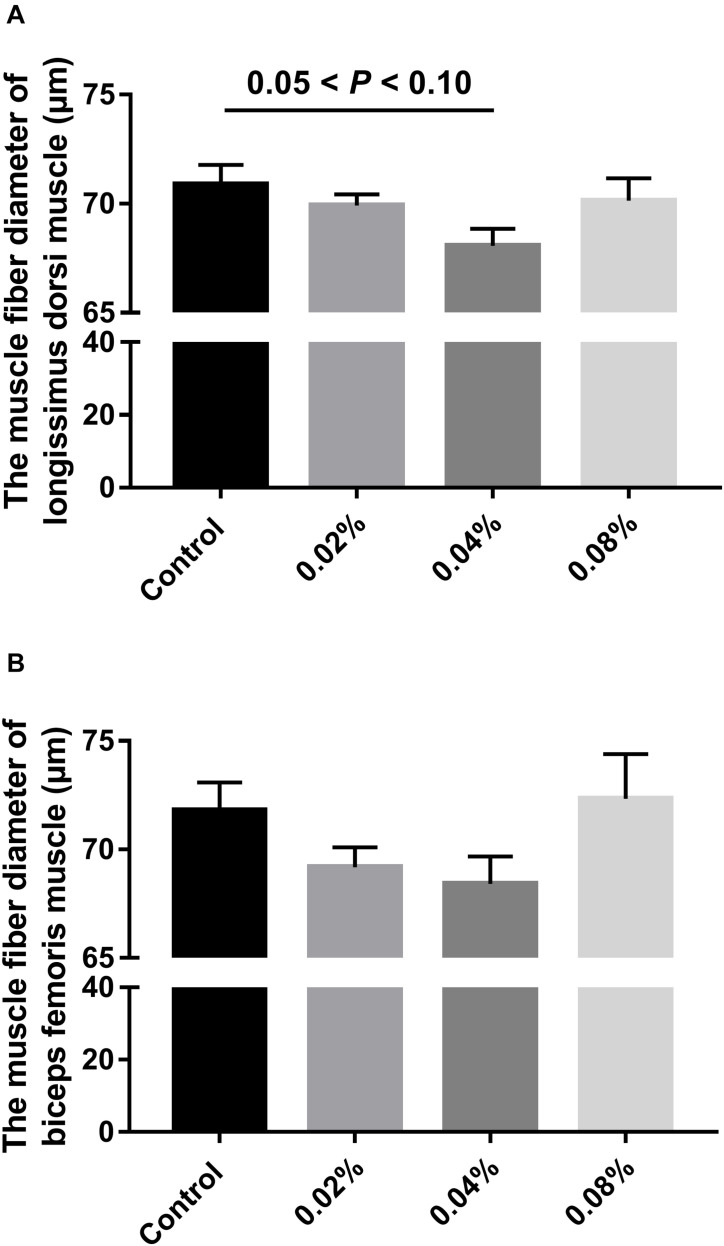
Effects of different levels of dietary CGA supplementation on muscle fiber diameter of finishing pigs. **(A)** The muscle fiber diameter of LD muscle. **(B)** The muscle fiber diameter of BF muscle. Values are means, with their standard errors represented by vertical bars.

Figure 2 summarizes the statistics regarding the muscle fiber diameter of finishing pigs fed with different levels of dietary CGA supplementation. No significant difference was found between groups (*P* > 0.05), although there was a tendency toward significance between the control group and the 0.04% CGA group (0.05 < *P* < 0.10).

### The Relative mRNA Expression Levels of Different Muscle Fibers and Myogenic Regulatory Factors

**FIGURE 3:**
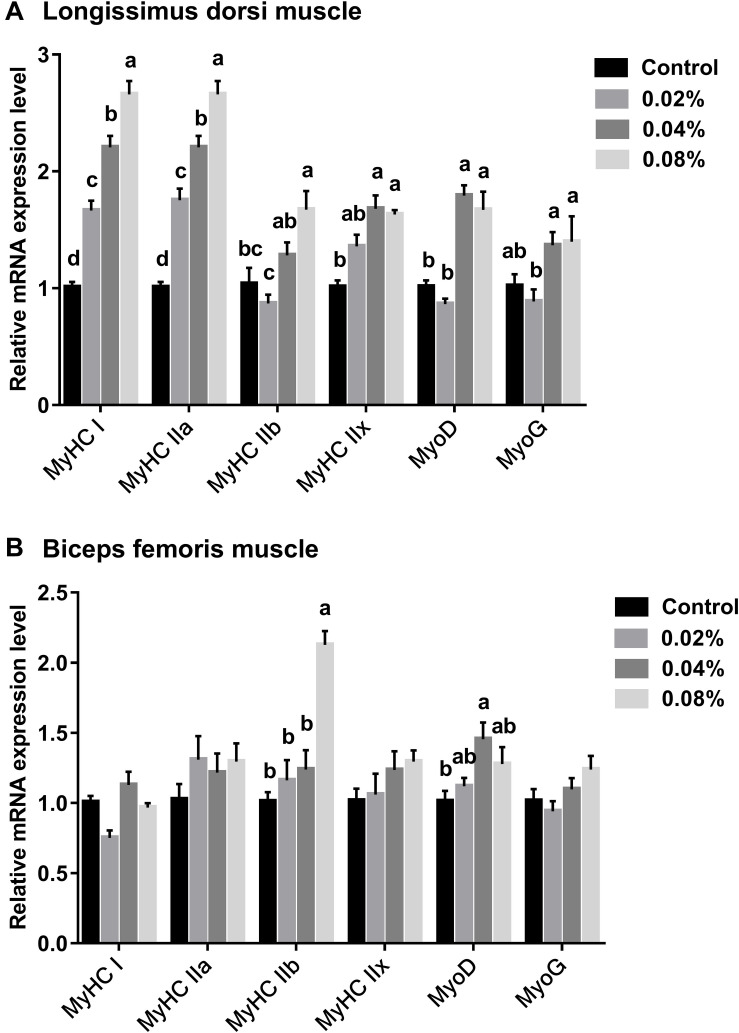
Effects of different levels of dietary CGA supplementation on relative mRNA expression levels of different muscle fiber and myogenic regulatory factors in LD **(A)** and BF **(B)** muscles of finishing pigs. Values are means, with their standard errors represented by vertical bars. Values with different letters (a–d) differ (*P* < 0.05).

The relative mRNA expression levels of myogenic regulatory factors and muscle fiber types were measured to assess the muscle fiber formation and characteristics of finishing pigs fed with dietary CGA supplementation. It can be visualized in Figure 3A that there is an increasing trend of relative mRNA expression of MyHC I and MyHC IIa in LD muscle of finishing pigs whose diet was supplemented with CGA (*P* < 0.05). Dietary supplementation with 0.08% of CGA resulted in a slight increase in the relative mRNA expression of MyHC IIb, which was significantly higher than that of the other groups (*P* < 0.05). In addition, compared with the control group, dietary supplementation with 0.04 and 0.08% of CGA led to an increase in relative mRNA expression of MyHC IIx (*P* < 0.05). Further statistical tests revealed that dietary supplementation with 0.04 and 0.08% of CGA significantly enhanced the expression of MyoD (*P* < 0.05). A slight increase in the expression of MyoG has also been recorded in the 0.04% and 0.08% CGA groups, but the differences were not significant (*P* > 0.05). Overall, these results indicate that dietary CGA supplementation improves the expression of myogenic regulatory factors and dramatically enhances the expression of oxidative myofibers (MyHC I and MyHC IIa) in LD muscle.

Dietary CGA supplementation has lesser efforts on BF muscle (Figure 3B). Notable differences in the relative expression level of MyHC IIb were found between the 0.08% CGA group and the other groups (*P* < 0.05). Furthermore, the 0.04% CGA group displayed a notable boost to the relative expression of the myogenic regulatory factor, MyoG (*P* < 0.05). Together, these results suggest that there is an association between dietary CGA and the expression of myogenic regulatory factors.

## Discussion

Evidence is building up to address the broad application prospects of CGA in the fields of medicine, food, and husbandry (Chen et al., 2018; Naveed et al., 2018; Lu et al., 2020; Papuc et al., 2020). Our experiment found that dietary supplementation with CGA contributed to the growth and carcass performance of pigs (data not shown), fitting well with a previous report using a finishing-pig model (Xu et al., 2019). Hence, this result establishes a factual basis for further research. To date, it has been well documented that a majority of natural plant compounds (including CGA) show a spectacular antioxidant capacity (Liang and Kitts, 2015; Hussain et al., 2021). Of note, the basic and most widely used biological property of CGA is antioxidant activity. In the current study, we determined the meat quality, nutritional values, and muscle fiber characteristics of finishing pigs to ascertain the relevance with the antioxidant property of CGA. As the most sensitive and rapid response to dietary CGA supplementation, the changes of the serum antioxidant parameters (especially GSH-PX) indicated the enlargement of antioxidant capacity. Moreover, the mRNA abundance of antioxidant-related genes in skeletal muscles confirmed the strengthening effect. However, it needs to be pointed out that over-supplementation of CGA may not enlarge even reverse the beneficial effects by causing unpredictable oxidative metabolic disorder. Overall, our data suggested that dietary supplementation with 0.04% of CGA gave the best improvement.

As the most widely consumed meat globally, the desire for high-quality porcine meat has grown with the continuously increasing demands for a higher quality of life. Meat quality is an essential economic trait within the swine industry. It is generally evaluated via physical attributes such as color, smell, taste, and tenderness by determining various intrinsic factors such as meat color, pH, drip loss, tenderness, intramuscular fat content, and chemical composition. Moreover, meat color is a vital factor in consumer purchase decisions since they associate the brown color with a loss of meat freshness and wholesomeness (Mancini and Hunt, 2005). Furthermore, as the most intuitive index to evaluate meat appearance and quality, meat color is primarily affected by the concentration of pigments, and secondly by the oxidation-reduction status and light-scattering properties of meats (Wu et al., 2015; Cooper et al., 2018; Keska et al., 2019). Pigments concentrating in meats include Myoglobin (Mb, 70–80%), hemoglobin (Hb, 20–30%), and colored metabolites (trace amount). Rather than the content, the properties of Mb play a crucial role in meat color determination (Suman and Joseph, 2013). It is known that the chromogenic effect of Mb relies on internal ferroheme, which has a strong affinity to oxygen. Thus, Mb can exist in any of three redox states: deoxymyoglobin (DMb), oxymyoglobin (MbO_2_), and metmyoglobin (MMb) (Mancini and Ramanathan, 2014). When it is not bound to oxygen, DMb presents as a dark red color. As a result, the formation of MbO_2_ (ferroheme combined with oxygen) is responsible for the consumer-desired bright cherry-red lean color (Mancini and Hunt, 2005; Tabke et al., 2017). Consumers usually discriminate against the presence of brown or discolored lean meat, a result of oxidation of MbO_2_ to MMb (caused by Fe^2+^ in heme oxidizing to Fe^3+^) (Bekhit and Faustman, 2005; Tabke et al., 2017). In general, when the flesh is observed to be dark brown with the naked eye, more than 60% of Mb has converted to MMb. Color coordinate values are recorded as L^∗^ lightness, a^∗^ redness, and b^∗^ yellowness. The L^∗^ value represents the lightness of meats, strongly associated with the amount of light absorbed by the pigments (including MB) in the meat (Purslow et al., 2020). The more light absorbed, the lower the L^∗^ value (Purslow et al., 2020). Regarding a^∗^ and b^∗^ values, they are both closely linked to the redox state of MB and the degree of conversion to MMB. Polyphenols like CGA can be oxidized enzymatically (e.g., catalyzed by polyphenol oxidases) or by autoxidation (e.g., in an alkaline environment or the presence of oxidizing agents) (Keppler et al., 2020). Thus, in the present study, dietary supplementation with 0.04% of CGA significantly decreased the b^∗^ value of LD muscle, indicating that CGA might reduce the formation of MMB by exerting antioxidant activity.

Inosinic acid (also called inosine monophosphate, IMP), succinic acid, glutamic acid, and some umami peptides are the main components responsible for meat taste and flavor (Zhang et al., 2008). Previous studies have demonstrated that IMP is an effective flavor enhancer, approximately 50-fold more effective than monosodium glutamate (Yan et al., 2018). Hence, IMP is a useful indicator of meat flavor worldwide (Suzuki et al., 1994; Lee et al., 2011). In this experiment, 0.04% supplemental dietary CGA resulted in a significant increase in the content of IMP in the LD and BF muscles. This finding is consistent with a previous study (Xu et al., 2019), in which an increase in the content of IMP in the muscles of finishing pigs fed with 800 mg/kg dietary apple polyphenol supplementation was recorded (CGA is one of the essential ingredients of apple polyphenol). The underlying mechanism is speculated to be connected with the fact that CGA can promote fatty acid β-oxidation and increase ATP production (Imafidon and Spanier, 1994; Zhang et al., 2010). More specifically, since the body’s normal physiological function is disrupted after slaughtering, the intracellular glycogen oxygen metabolism transforms into anaerobic glycolysis. Subsequently, the production of lactic acid lowers the pH in muscles, and the decrease of ATP in muscles leads to the dysfunction of the sarcoplasmic reticulum. Meanwhile, ATPase activity is therefore rapidly activated, promoting the decomposition of the remaining ATP to ADP. Due to the activities of various enzymes, IMP is produced and further hydrolyzed to form hypoxanthine and ribose. Thus, the presence of CGA enhances the umami taste of meats by triggering accumulation of IMP and other breakdown products in muscles.

Since amino acids are the basic units of proteins, the composition of amino acids is an important indicator used to evaluate the quality of the protein and the nutritional value of pork (Wood et al., 1996; Maggiolino et al., 2020). In the present study, the results showed that the amino acid composition within LD and BF muscles was drastically changed. 0.04% supplemental dietary CGA induced an increase in the proportions of flavor amino acids (FAA) such as Arg, Ala, Asp, Glu, and Gly in muscles to varying extents. These amino acids are typically considered related to the umami taste of pork and can react with soluble reducing sugars, such as glucose and fructose, to form flavoring substances (Imafidon and Spanier, 1994; Zhang et al., 2010; Yu et al., 2012). Amino acids in pork are also essential from a nutritional perspective, especially the essential amino acids (EAAs), which the human body cannot synthesize and need to obtain from diets (Mediani et al., 2017). In this experiment, we observed that 0.04% dietary CGA supplementation initiated a mild and an intense augmentation of EAAs content in LD and BF muscles, respectively. This outcome implies that adding 0.04% CGA to the diet of the finishing pigs is beneficial to the flavor and the nutritional value of pork.

Previous researches indicate that some polyphenol-enriched plant extracts have potential anabolic effects that could promote myogenesis and anti-catabolic effects on muscles (Jang et al., 2018; Alsolmei et al., 2019). To figure out whether CGA has these functions, we measured the mRNA expression levels of different muscle fiber types and critical myogenic regulatory factors in the finishing pigs fed with dietary CGA supplementation. Interestingly, we obtained new data that support this opinion. Our results demonstrated that 0.04% dietary CGA supplementation notably upregulated MyoD mRNA expression and mildly elevated MyoG mRNA expression in both LD and BF muscles. It has been well documented that myogenic regulatory factors including Myf4/5, MyoD, and MyoG serve a crucial function during developmental myogenesis and myogenic differentiation (Hamed et al., 2017; Zammit, 2017). MyoD, in particular, is regarded as a crucial “master switch” in regulating muscle-specific gene transcription involved in muscle protein synthesis (Shintaku et al., 2016; Ding et al., 2019). Thus, we report that CGA might promote myogenesis by upregulating the expression of myogenic regulatory factor MyoD. Additionally, another study using cultured cells and an adult skeletal muscle model confirmed that MyoD regulates oxidative metabolism (Shintaku et al., 2016). Taken together, this implies the possibility that the promotion of myogenesis by CGA might be associated with its antioxidant activity. However, despite these promising results, the underlying metabolism remains unclear. Therefore, further studies on this topic are recommended.

Numerous studies have reported that muscle fiber characteristics and antioxidant capacity are associated with various meat quality traits such as muscle pH, tenderness, drip loss, and meat color (Zhang et al., 2015; Li et al., 2018). Skeletal muscles are composed of four specific myofiber types: oxidative (I and IIa), intermediate (IIx), and glycolytic (IIb) (Duan et al., 2017a). Different skeletal muscle fiber types are reported to exhibit distinctively different physiological and metabolic properties (Percival and Froehner, 2007; Zhou et al., 2016a; Li et al., 2020). A previous study has illustrated that muscle fibers are not static structures and quickly adapt to altered environmental factors, such as changes in nutritional input (Duan et al., 2017b). Concrete evidence has been reported that a muscle fiber type transition toward more oxidative muscle fibers such as MyHC I can be induced by antioxidants (Liu et al., 2008). Interestingly, our data herein is consistent with their conclusion. 0.04% dietary CGA supplementation greatly enhanced the mRNA expression levels of the oxidative muscle fiber types (I and IIa) in LD muscle. As for the observed discrepancy regarding the effects of dietary CGA supplementation on LD and BF muscles, a possible explanation may be that the proportion of oxidative and glycolytic myofiber types in LD and BF muscles are different (Li et al., 2016). Usually, LD muscle has a higher percentage of glycolytic fiber (IIb) than BF muscle (Li et al., 2016; Duan et al., 2017b), thus react more intensively to antioxidants. This finding has important implications for the potential action of dietary CGA supplementation altering muscle fiber characteristics of finishing pigs by exerting its natural antioxidant capacity.

## Conclusion

In summary, this study demonstrates that 0.04% of CGA supplemented in the diet of the finishing pigs can improve meat quality, including meat color, meat flavor, and nutritional values, thereby favoring the utilization of natural waste substances derived from herbal plants. Moreover, this work also provides evidence in pigs that CGA induces a shift toward more oxidative muscle fibers and increases antioxidant capacity. Despite its exploratory nature, the present experiment offers some insight into the beneficial effects of natural plant extracts on meat quality. These findings provide a useful reference for the development of future industrial swine practices.

## Data Availability Statement

The original contributions presented in the study are included in the article/supplementary material, further inquiries can be directed to the corresponding author/s.

## Ethics Statement

The animal study was reviewed and approved by the Animal Care Committee of the Institute of Subtropical Agriculture, Chinese Academy of Sciences (Changsha, China).

## Author Contributions

WW and YY designed the experiments. WW conducted most of the experiments, analyzed the data, and wrote the manuscript. CW and QG helped to collect the samples and conduct the experiments. JL and YY supervised the study, designed the experiments, and revised the manuscript. SH contributed to revising the manuscript. All authors read and approved the manuscript.

## Conflict of Interest

The authors declare that the research was conducted in the absence of any commercial or financial relationships that could be construed as a potential conflict of interest.
